# Influence of zonular tension on molecular transport in the porcine ocular lens

**DOI:** 10.3389/fopht.2024.1508779

**Published:** 2024-12-02

**Authors:** Morgan Crews, Wade Rich, Matthew A. Reilly

**Affiliations:** ^1^ Department of Biomedical Engineering, The Ohio State University, Columbus, OH, United States; ^2^ Department of Ophthalmology and Visual Sciences, The Ohio State University, Columbus, OH, United States

**Keywords:** crystalline lens, tension, diffusion, stretching, lens circulation

## Abstract

**Introduction:**

Accommodation is the process of changing the ocular lens’ refractive power and focal distance. This process involves application of biomechanical forces on the lens by the surrounding musculature. Previous studies have demonstrated that the lens epithelium demonstrates mechanotransduction and that tension influences its chemical activity. It is not yet known how these forces affect the structure and permeability of the lens. This study aimed to identify the influence of tension on molecular transport of dyes through the lens.

**Methods:**

Paired porcine eyes were incubated in each of four dyes for three time periods with no stretch (null), static, or cyclic stretching using a bespoke mechanical lens stretcher. After incubation, the lenses were frozen and cryosectioned sagittally through the optic axis. Photographs of the stretched and unstretched lenses were compared and qualitatively assessed.

**Results:**

None of the four dyes showed drastic stretch-induced differences in dye penetration depth. However, the dye neutral red showed dramatic stretch-induced changes in the dye uptake color behind lens anterior surfaces, with unstretched lenses appearing far more orange than their stretched counterparts. Three of four dyes showed notable differences between anterior and posterior diffusion patterns. One dye, methylene blue, demonstrated unexpected intensity in the lens nucleus compared to the lower intensity shown in the cortex, suggesting active transport rather than a linearly graded passive diffusion regardless of stretching condition.

**Discussion:**

All this taken together suggests that lens transport is more complex than simple passive diffusion and that active transport of some molecules may be affected by stretching. Future work should assess the mechanisms of transport for the various dyes and attempt to explain the dye permeation patterns observed here, including the effects of stretching.

## 1 Introduction

**Figure 1:**
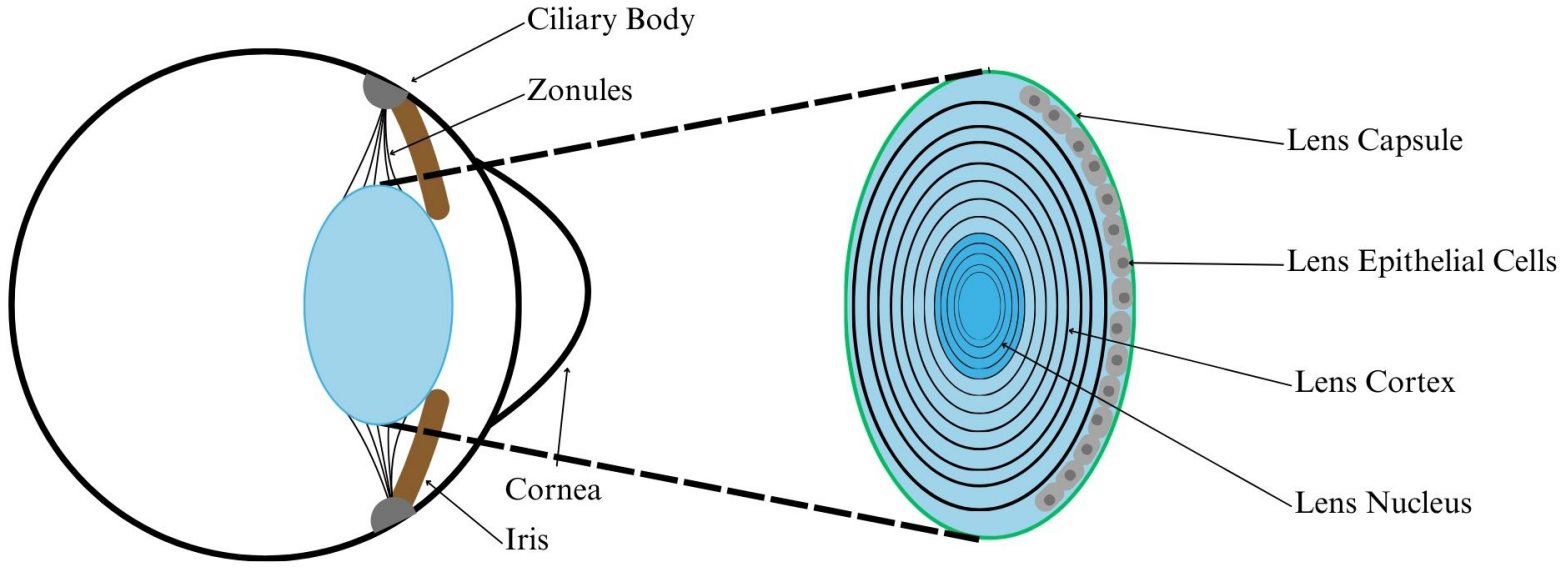
Diagram showing ocular lens anatomy. The lens is comprised of many layers of transparent matrix, with metabolically active lens epithelial cells along the anterior surface. The lens is held in place behind the iris by zonules, which are connective fibers extending from the ciliary body. The ciliary body contains the ciliary muscle, which exerts force on the zonules and lens capsule to change the shape of the lens.

Accommodation is the ability of the crystalline ocular lens to change shape, resulting in a wider range of focal distances. During accommodation and disaccomodation, the ciliary muscle constricts and relaxes which modulates tension in the zonular fibers that connect the lens to the ciliary body and attached sclera (**Figure 1**). Recent studies have indicated that the causes and/or treatments of multiple lenticular pathologies, such as Presbyopia and cataract, are likely to be biomechanical in nature (1–6). There are no efficient preventative therapies for these pathologies, and current treatments can only help symptoms rather than restore accommodative function. A more comprehensive understanding of ocular biomechanics will reveal the root causes of lenticular disease, lead to effective preventative treatments, and improve the accuracy of ocular modeling.

The optical changes caused by accommodation can be mimicked ex vivo by varying radially applied mechanical forces (1–8). While there have been many variations in the methodology, all studies use the principle of radial stretching and transmission of forces through the zonules onto the lens capsule. The lens capsule is a basement membrane composed of extracellular matrices of lens epithelial cells (LECs) and has been shown to exhibit mechanotransduction (6). When force is applied through the zonules, the lens capsule, deforms the lens fiber cells changing the curvature of the biconvex lens (9). The mechanical properties of the lens capsule are also yet to be fully illuminated.

The chemical properties of the lens capsule have been more thoroughly studied than the lens mechanical properties. The lens capsule is known to be permeable to small molecules such as water, carbon dioxide, and oxygen (10, 11). Studies have additionally shown the lens capsule has selective permeability to nutrients and proteins needed for cortical fiber cells development and metabolism (9, 12, 13). The current understanding in the field is that lens capsule permeability varies greatly depending on molecule size, polarity, hydrogen bond affinity, and other chemical properties (12–15). Additionally, zonular tension has been shown to influence both aquaporin activity and hydrostatic pressure gradient, which are intrinsically linked to lens capsule properties (16).

The geometry, physical properties, chemical properties, and tissue composition of the anterior and posterior lens differ significantly. The anterior lens houses a monolayer of LECs that are most metabolically active just anterior of the equator where LECs continually proliferate throughout life. The lens is biconvex, meaning that each side has a distinct convexity throughout all accommodative conditions (17). The posterior has a larger curvature, resulting in larger lens volume posterior to the equator.

In 1997, Mathias et al. proposed the lens Fluid Circulation Model (FCM), which theorized a pattern of transport going inward at the poles and outward at the equator (18, 19). Since then, multiple studies have supported this model, and it is becoming foundational in lens transport research (20–23). It has yet to be investigated whether this pattern of microcirculation holds true for all molecules or in various accommodative/stretched conditions.

It is thus hypothesized that LEC mechanotransduction and chemical effects of tension are likely to be observed after the lens is exposed to tensile stretching force. Few studies to date have analyzed the effects of stretching on transport in the lens. In theory, application of mechanical force will alter the structure of the lens capsule and thus influence permeability. Stretching could also affect permeability of LECs and fiber cells even deep within the lens cortex. Variations in dye permeation under different stretching conditions may elucidate the structural behavior of the lens capsule and underlying cells under accommodative conditions.

## 2 Materials and methods

**Figure 2:**
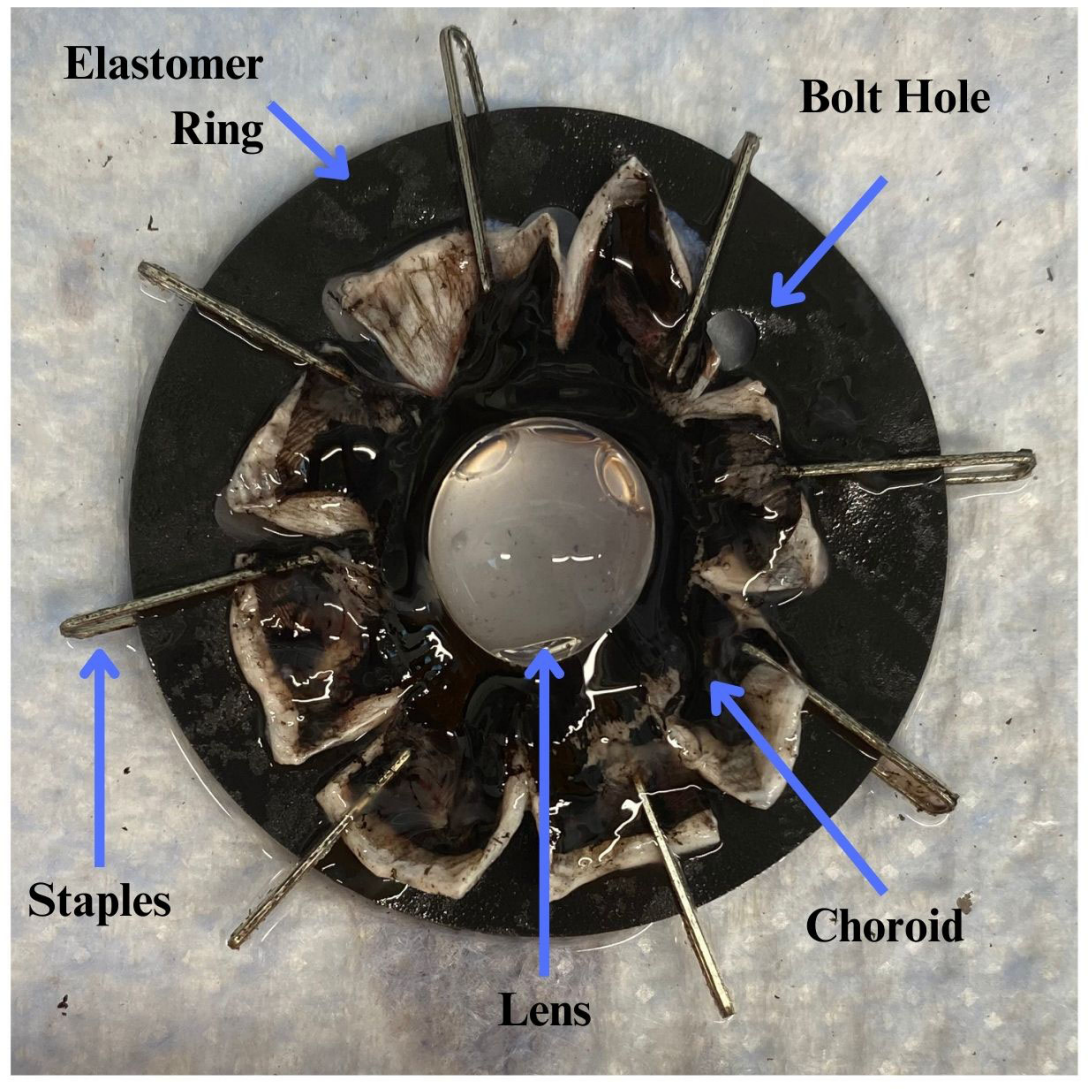
After the porcine eyes were cleared of cornea, iris, and vitreous humor, they were bisected and the posteriors discarded. The sclera was then cut into 8 flaps to allow the anterior cup to lay flat. The equatorial sclera was stapled to a silicone elastomer ring via 8 equally spaced staples. The ring’s 8 bolt holes were used to affix the sample to the stretching device. The sample photo was oriented with its anterior facing into the page and posterior lens closest to the reader.

Fresh, paired porcine eyes were obtained from a local abattoir. Pig sex was unknown, and age was 6 months. The lenses were dissected less than four hours after the whole eyes were extracted to maximize tissue viability. The dissection involved removing the cornea, iris, vitreous humor, and the posterior sclera (6). The zonules and ciliary body were left intact to allow force distribution onto the lens capsule. The anterior cup (sans cornea, iris, and vitreous humor) was mounted to a silicone elastomer ring via staples in the equatorial sclera, as shown in **Figure 2**.

**Figure 3:**
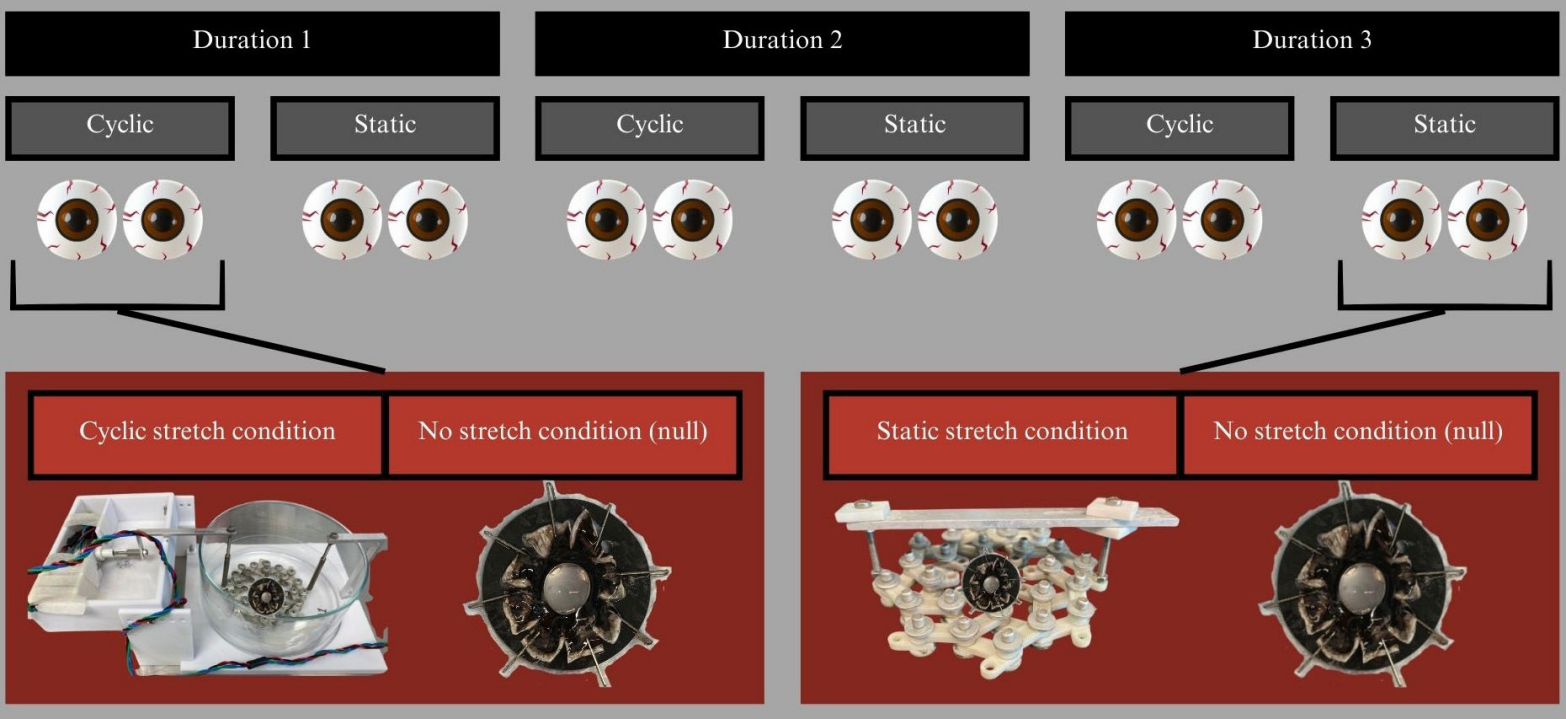
Experimental design showing how paired eyes were divided into experimental conditions. Each dye was incubated for 3 time points and each time point had a cyclic and a static experiment. Each stretch condition had one eye of the pair stretched and the other not. This system was replicated for 4 dyes, though one of the dyes could not survive the 2^nd^ or 3^rd^ time point.

**Figure 4:**
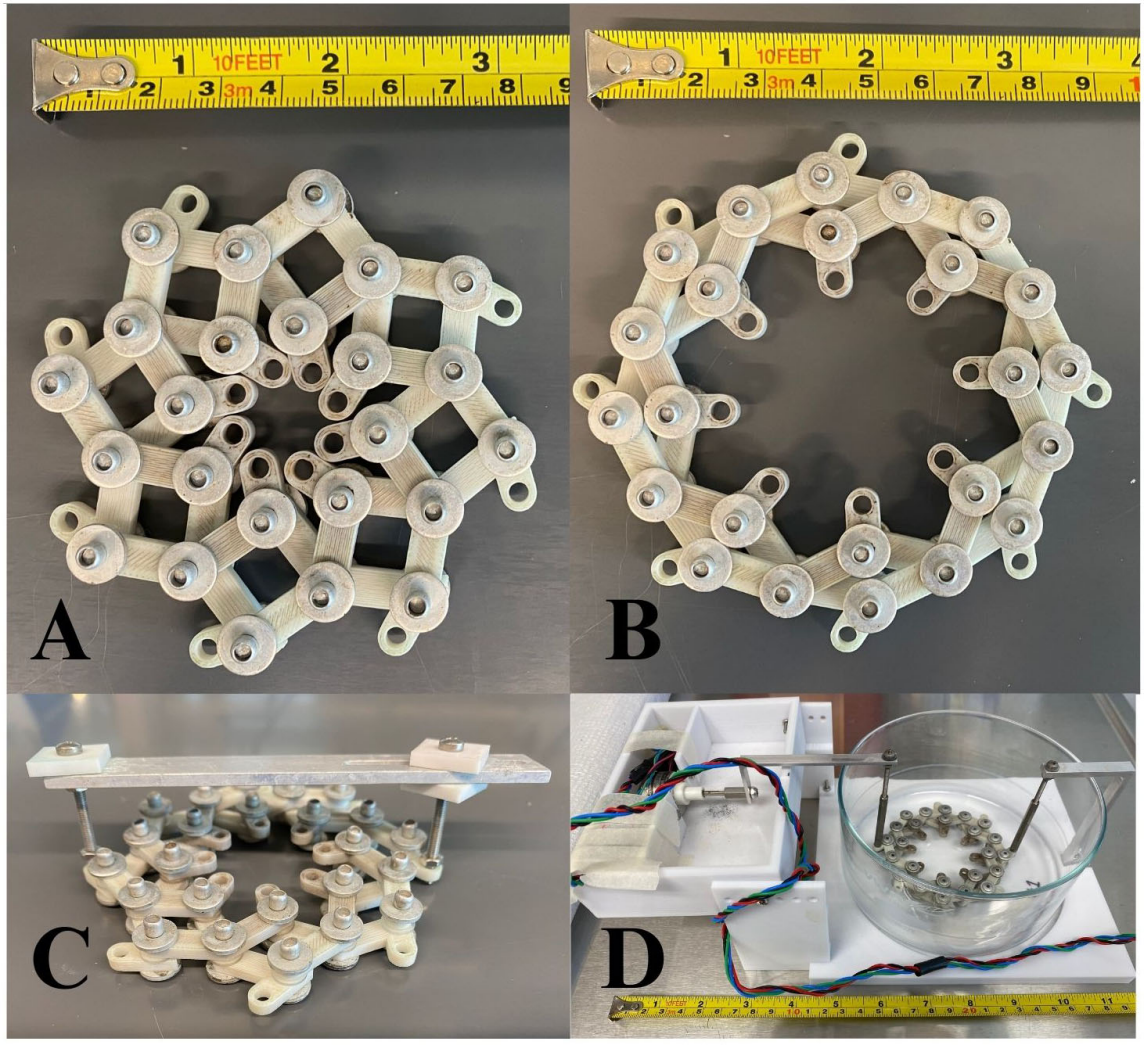
**(A)** Bespoke stretching apparatus in the unstretched and **(B)** stretched positions. The eye was stapled to an elastomer ring, which was bolted into the center of the device. Force on the stretcher’s outer edge caused the sample to be stretched equally in all directions, similarly to accommodation. **(C)** Stretcher in its static stretching apparatus. The metal bar across the top held opposite sides of the stretcher a fixed distance apart to continuously stretch the sample in a radial fashion. The whole stretcher and its sample were submerged in a large dish of dyed media. **(D)** Cyclic stretching apparatus inside its glass dish for submersion in media and dye. The right side of the stretcher was affixed to the plastic base plate while the left side was attached to an Arduino-powered oscillating motor.

The paired eyes were incubated with 4 different dyes, 3 durations of incubation, and one of three stretching conditions: cyclic, static, or control (null), as shown in **Figure 3**. The silicone rings attached to experimental eye cups were attached using 8 bolts to the stretching apparatus shown in **Figure 4**, which allowed for equally distributed stretching along the eye cup’s circumference (6). A single motor was used to impart equibiaxial stretching using a 3D-printed lens stretcher as discussed further in 6. The statically stretched eye cups were placed in the stretcher and the equatorial radius increased by 6-12% using the rigid bar shown in **Figure 4C**. The cyclically stretched cups were connected to the stretcher in the same manner (**Figure 4D**). The cyclic stretcher was then fixed on one side and attached to an Arduino controlled motor on the opposite side. The motor caused the stretcher to continually oscillate between the stretched and unstretched positions throughout the incubation period at about 0.25 Hz.

Once set up in their respective stretching apparatus, each system was placed in a 10:1 media to dye solution covering the entire eye cup and put in an incubator at 37°C and 5% CO2. This experiment used four dyes, chosen to represent a variety of molecular weights and hydrogen bond affinities, which were Neutral Red (NR) (Sigma Aldrich, 72210-5G), Methylene Blue (MB) (Millipore Sigma, M4159-25G), Crystal Violet (CV) (Sigma Aldrich, 61135-25G), and Erythrosin Extra Bluish (EB) (Sigma Aldrich, 45690-10G-F). **Table 1** shows some relevant properties of each dye. All dyes were used at a concentration of 1mg dye powder to 1mL deionized water and 1mL liquid concentrate to 10mL media. All tests used media 199 without phenol red to eliminate confounding color effects (Gibco, 11043-023). Each stretch/dye combination was incubated for 3 time periods before imaging. Eyes were incubated for 24, 48, and 72 hours for MB, CV, and EB dyes. In preliminary testing, NR had a particular affinity for the lens cortex and was therefore incubated at 1, 3, and 6 hours.

**Table 1:** Chemical properties of 4 dyes used.

	H-bond Acceptor	H-bond Donor	Molecular Weight (g/mol)	Polar Surface Area (Å^2^)
Methylene Blue	4	0	319.9	43.9
Crystal Violet	3	0	408	9.5
Neutral Red	4	2	288.77	55
Erythrosin Extra Bluish	5	0	879.9	81.6

**Figure 5:**
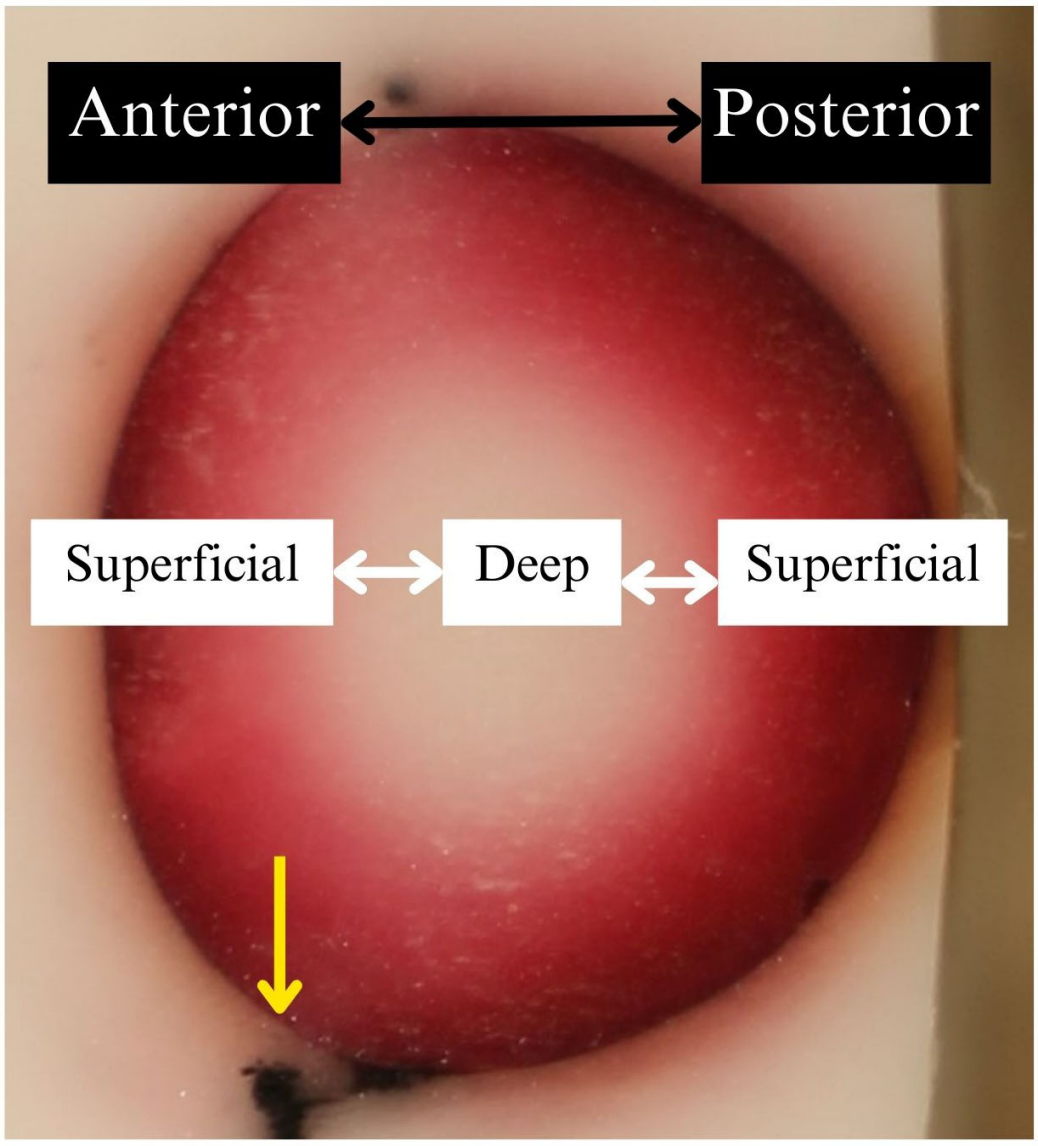
Representative experimental result of a lens embedded in frozen OCT compound after submersion in dyed media. Photos were oriented with the anterior facing left and superficial aspects closest to the photo’s edges. The lighter colored/white background was frozen OCT. The brown line was the outline of the lens, most likely caused by dye particles adhering to the outer surface of the lens capsule. The center of the photo showed dye uptake in the lens cortex via color intensity. The yellow arrow indicates black zonule tissue that remained at the lens equator.

Once incubated, the lenses were removed from the eye cups and frozen in Optimal Cutting Temperature (OCT) compound for at least 12 hours at -80°C. The OCT Blocks were mounted into a cryotome and cut to reveal sagittal planes of the lenses. The sagittal midline or optic axis was assumed to be the cross section that had the largest area within the lens outline. Photos were taken using a dSLR camera at a standardized angle and distance (**Figure 5**). Photographs were organized into figures to allow direct visual comparison between experimental conditions within each dye.

## 3 Results

**Figure 6:**
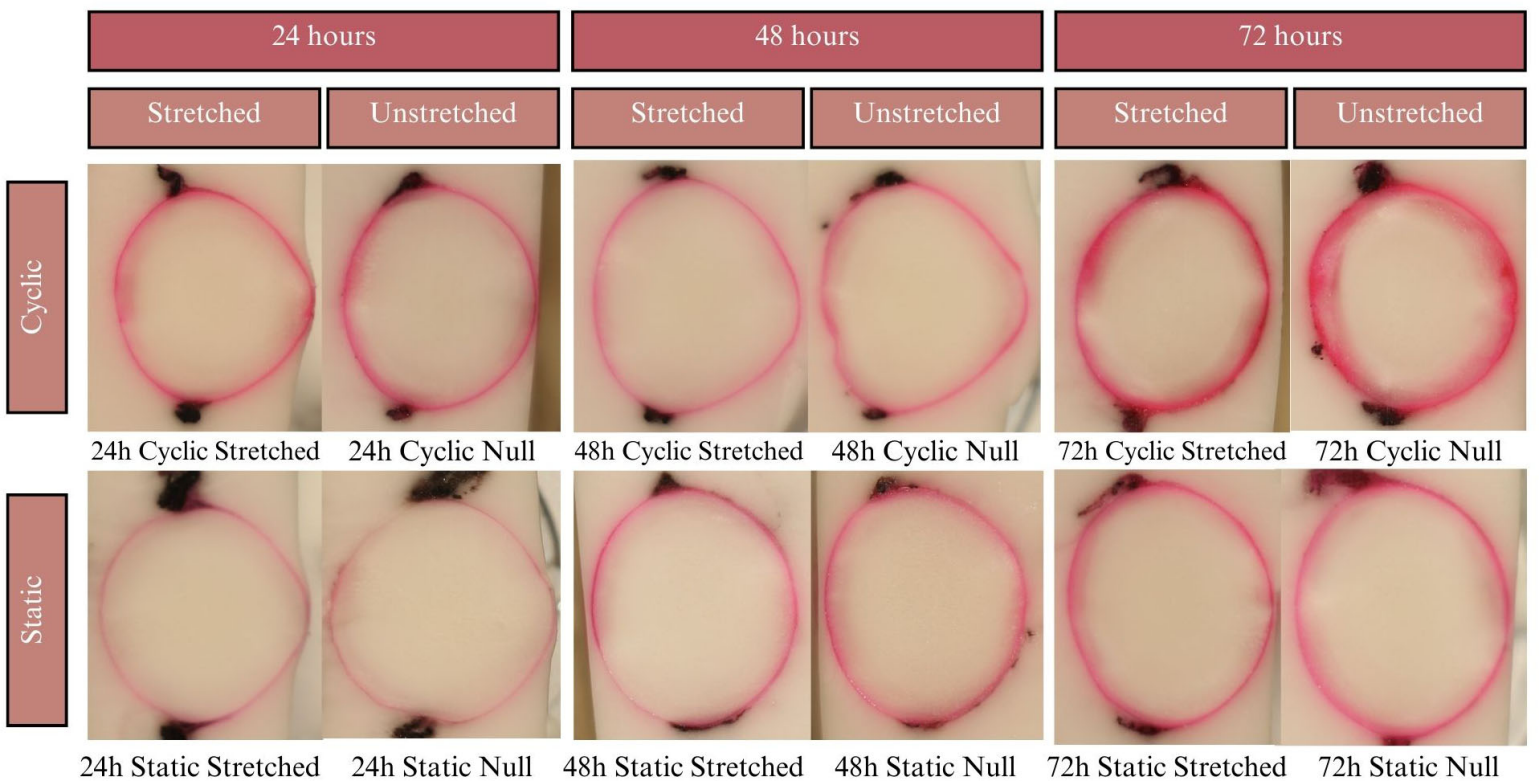
Results of the EB experiments after 24, 48, and 72 hours of incubation. Dye uptake manifests as bright pink, while freezing medium and areas of no dye uptake appear white. The pink outlines of the lenses are relatively thin, covering only the lens capsule with little to no penetration into the cortex.

The cryotome sectioning process yielded images like those seen in **Figure 6**. All photos were oriented with the anterior lens toward the left and the posterior lens toward the right.

**Figure 7:**
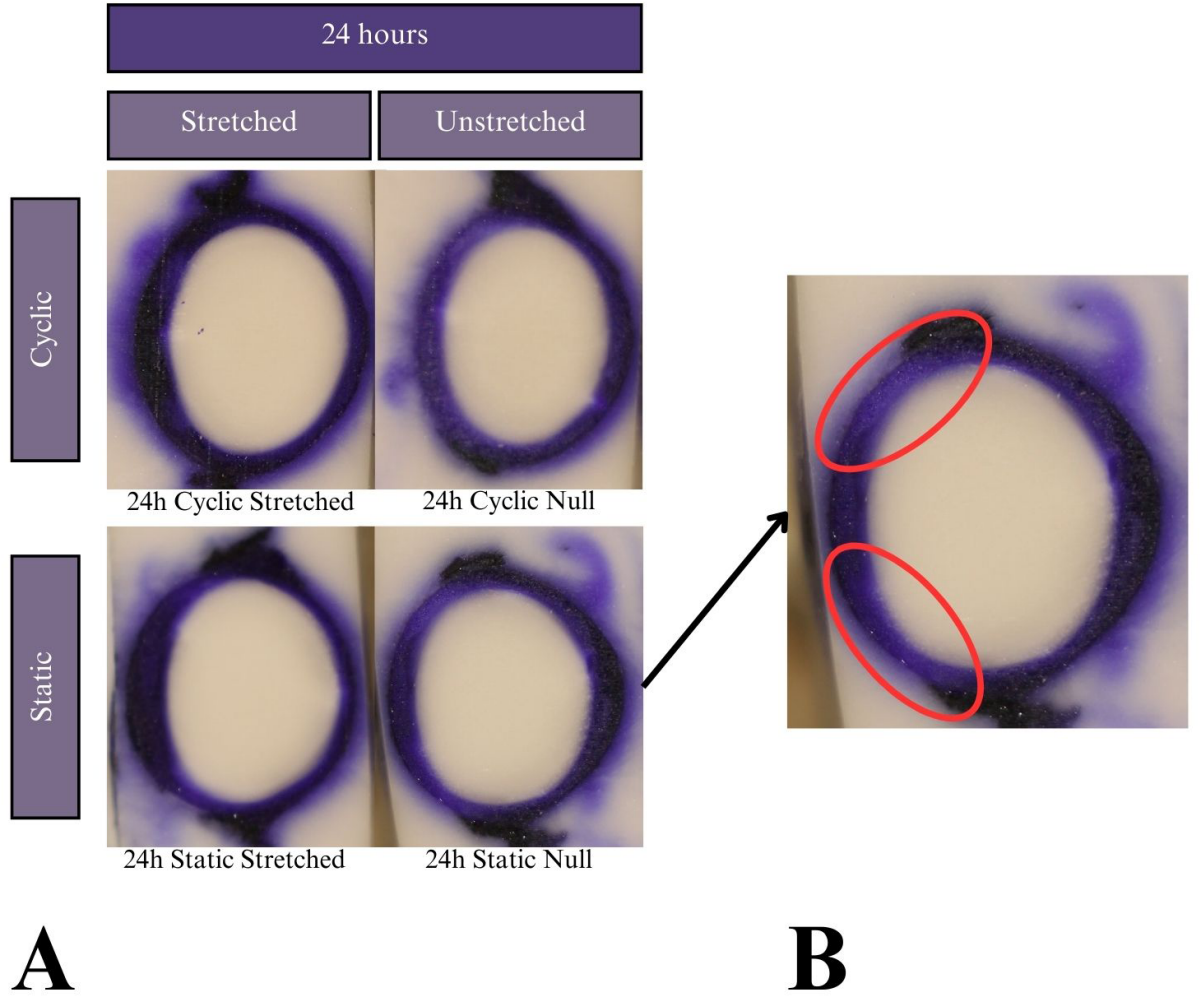
**(A)** Results of the CV experiments after 24 h of incubation. Tissue samples did not keep tension for 48- or 72-hour tests, so the results are not reported. Dye uptake manifests as dark purple, while freezing medium and areas of no dye uptake appear white. **(B)** Expanded image of the 24 hour-incubated null eye of the static test. Red ovals indicate areas of lower dye uptake on the lens anterior towards the equators compared to higher uptake on the anterior pole.

**Figure 8:**
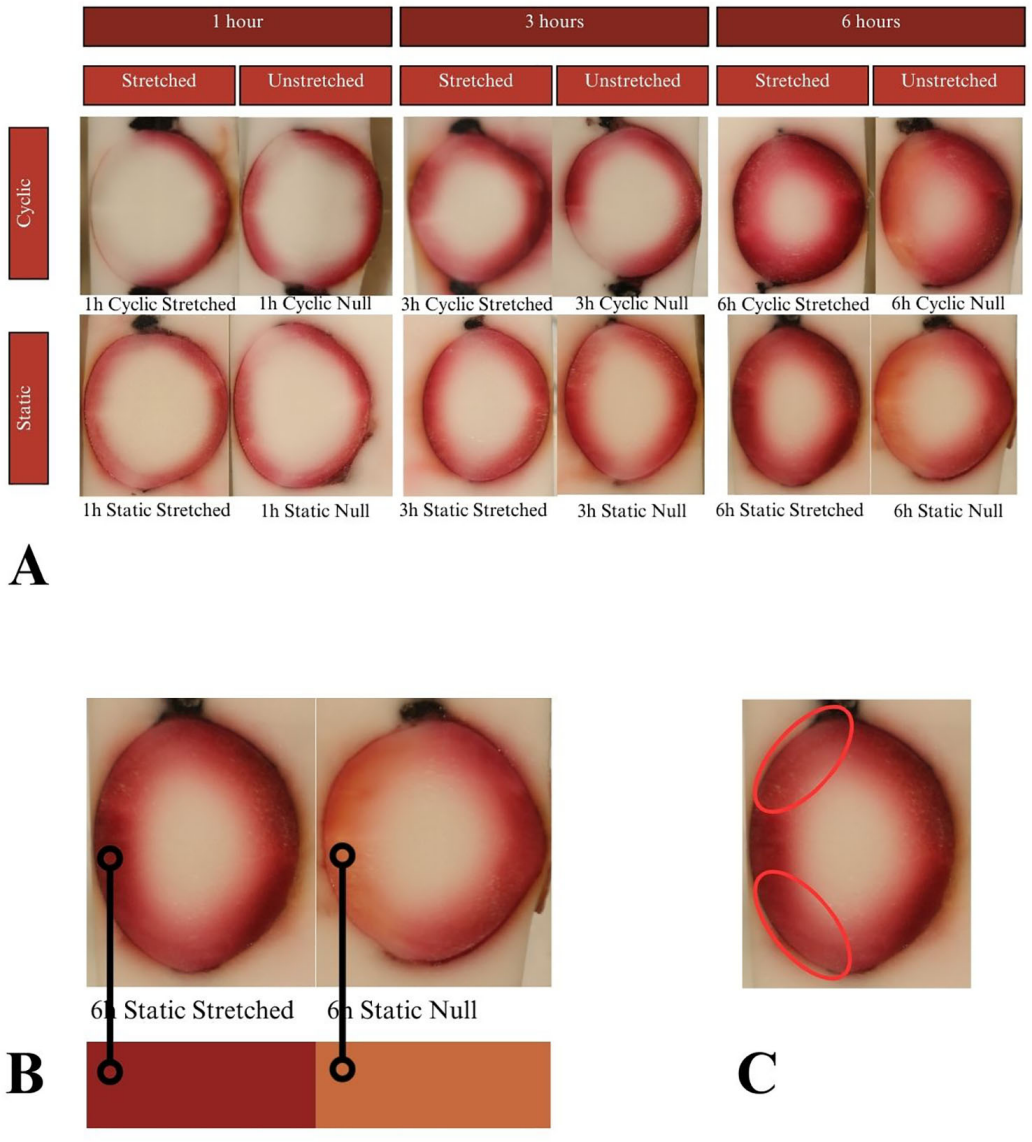
**(A)** Results of the NR experiments after 1, 3, and 6 hours of incubation. Dye uptake manifests as red, while freezing medium and areas of no dye uptake appear white. All photos show simple linear gradients appearing the darkest at superficial areas and decreasing intensity going deep. **(B)** Analysis of 6-hour NR samples. The anterior sides of the unstretched samples showed a more orange color compared to their own posterior sides and compared to the paired anteriors. NR being a pH indicator, this result suggests that stretching has a physiological impact on acidity. **(C)** All samples showed regions of decreased intensity at the anterior equators, which are known to be LEC proliferation sites.

**Figure 9:**
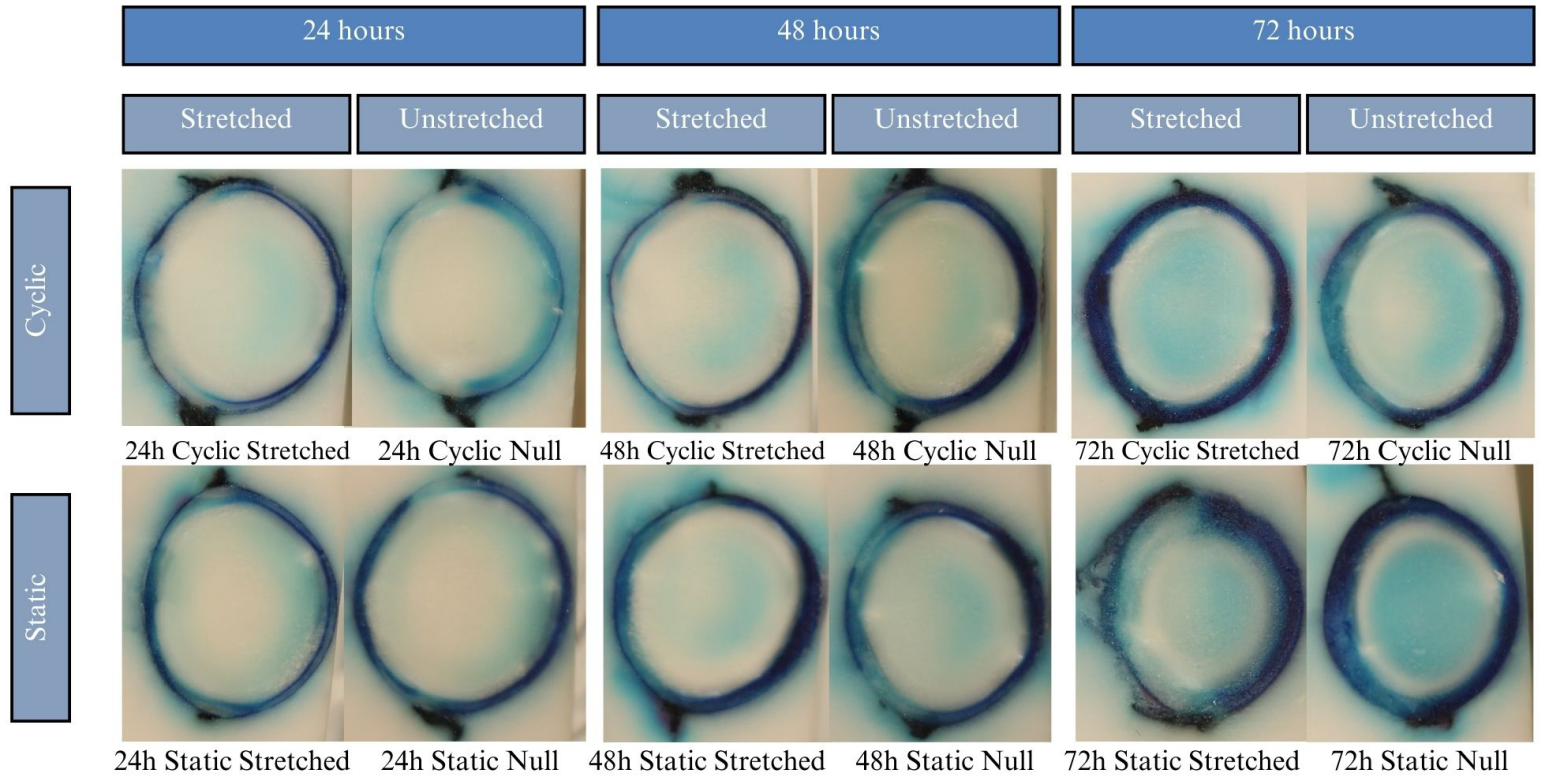
Results of the MB experiments after 24, 48, and 72 hours of incubation. Dye uptake manifests as blue, while freezing medium and areas of no dye uptake appear white. All samples show a dark ring deep to the lens outline, which abruptly decreases intensity in the lens cortex. Samples incubated for 48 hours show a mild patch of dye uptake in the lens center. For 72-hour samples the central mass of dye is darker and more heavily contrasts with the white just superficial to it. This pattern of nonlinear dye uptake indicates that lens microcirculation of MB is influenced by active transport rather than mere passive diffusion.

Lenses were incubated for up to 24 hours in CV, but beyond that showed considerable degradation of the zonules, in all cases causing detachment of the lens from the sclera. Due to zonular failure, this subset of CV lenses was unable to be assessed. The results of the 24-hour duration can be seen in **Figure 7A**. Lenses were incubated for 24, 48, and 72 hours in solution containing EB. As seen in **Figure 6**, EB did not permeate past the lens capsule even at the longest incubation duration. The EB samples did not show any visual differences between stretch conditions or anterior vs. posterior surfaces. The intensity of the dye adherence to the superficial lens capsule was, as expected, greater after longer exposure. The concavity of the anterior and posterior lenses was as expected and relatively consistent. In preliminary experiments, NR dye showed a much higher affinity for the lens than other dyes, such that the lens was fully colored before 24 hours. This led to alteration of the incubation duration to 1, 3, and 6 hours. As seen in **Figure 8A**, NR experiments showed the expected gradual increase in concentration over time and the expected intensity gradient going from superficial penetration to complete penetration into the lens nucleus. Differences between stretch conditions were not perceptible for 1 or 3 hours. However, for both cyclic and static stretching, lenses stretched for 6 hours showed different coloring on the anterior side when compared to their unstretched controls. As seen in **Figure 8B**, unstretched NR 6h lens anterior regions were noticeably more orange than unstretched NR 6h lens posteriors, unstretched NR lenses at all other time points, and stretched NR 6h lens anterior sections. MB did not show consistent observable changes between stretching conditions (**Figure 9**). The overall presence of dye was greater at longer time points, as expected, but its distribution pattern was distinct from other dyes. While NR showed a high intensity superficially which decreased gradually as depth increased, MB exhibited a more piecewise distribution of dye. The outermost ring of tissue had a sharp rather than gradual drop in intensity, then a ring of little to no dye uptake, and finally a moderate intensity in the deepest region. There existed a non-monotonic concentration gradient within the lens. Nine of twelve MB samples showed disparity in anterior-posterior dye distribution. Posterior sides of both the whole lens and the nucleus tended to have higher intensity concentration than the respective anterior.

Additionally, MB seemed to induce shape changes in the lens, making its overall shape more ovular than the typical biconvex. Future work should analyze the curvature changes quantitatively and elucidate the reasoning for such changes.

## 4 Discussion

The mechanism by which the zonules degraded in CV solution was not evident in this study’s results nor the literature due to CV’s novel use in the lens. Since the tissue cannot be cultured beyond 24 hours with CV, it is not an ideal dye to use in lens stretching research. Notably, all samples showed a slightly decreased permeation on the anterior surface closest to the equator, as shown in **Figure 7B**. This may be due to the lens anterior having a layer of lens epithelial cells (LECs) that is known to be metabolically active.

EB’s lack of penetration could be due, in part, to its large polar surface area, which indicates the molecule is highly polarized. More importantly, EB molecules have a molecular weight of nearly 880 g/mol, which is 2-3 times as large as the other dyes. This shortcoming prevents any visualization of changes from stretch condition, and as such we were able to determine that EB is an unsuitable dye with which to visually assess lens transport in these culture conditions.

NR is known to act as a pH indicator from 6.8 to 8 as red to yellow, respectively (Sigma Aldrich n.d.). This indicates unstretched NR 6h anteriors had a higher pH and lower acidity than all other test conditions. The mechanism by which stretching preserves the physiological pH in this case is unknown and warrants further inquiry. Perhaps LECs are dependent upon a certain level of tension to remain healthy, and absence of tension led to a buildup of acidic byproducts which manifested in the NR uptake color. This correlation between tension and lens activity would be a natural sequitur from the research suggesting LECs experience mechanotransduction. Additionally, NR lenses showed a similar decrease to CV in absorption at the areas of active cell proliferation on the lens anterior, again suggesting metabolic influence.

The lower permeation of dye at the LEC proliferation sites seen in CV and NR (**Figures 7B**, **8C**) could be due to several factors. The metabolic activity itself or the phenotype of the LECs could have resulted in less uptake. Alternatively, these cells may have absorbed the same amount of dye but processed or degraded it more quickly or more thoroughly than other tissues did. A difference in molecular breakdown chemistry may also relate to the observed color change between NR 6h stretched vs. unstretched.

MB’s irregular concentration gradient strongly supports that the lens has pathways of active transport for certain molecules and does not merely rely on passive diffusion. The increased central concentration of MB seems to occur within the outline of the lens nucleus, which is of a much higher cell density than the surrounding cortex. The ring of low dye concentration between the outer cortex and the outer nucleus defies the expected gradation pattern. That is, where we expect the lens to be darkest on the periphery and lightest in the innermost region, it appears lightest in the central concentric layer and moderate at the nucleus core. Standard fluid dynamics declares that unaided diffusion occurs in a concentration gradient going predominantly from areas of high to low concentration, which we do not see here. As such, a more complex process like active transport must be at work. The pathways illustrated by the dye concur with predicted models of lens diffusion. Full analysis of the chemical differences between the dyes and how this manifested in differing patterns was outside the scope of this paper but should be further explored.

MB samples containing higher posterior concentrations is unusual due to the posterior’s larger area, which intuitively should result in a more diffuse dispersion. This result may suggest that the epithelium of the anterior provides a protective barrier against certain molecules used in this study.

CV and EB were ineffective at visualizing lens transport, in this particular application, due to induced tissue degradation and low penetration depth, respectively. NR dye showed stretch-induced pH differences between anterior and posterior surfaces at 6 hours. Future work should inspect the mechanism of this pH change and how stretching contributes. MB demonstrated clear pathways of active transport with significant anterior-posterior differences, though stretching effects were not apparent within this sample. These findings are in agreement with the microcirculation or FCM by Mathias et al., though CV and MB require more chemical characterization in the lens to be situated in the literature.

This study was limited by both small sample size and lack of statistical quantification. Quantification is possible using image analysis software to convert RBG images into vectors of intensity values, which correlate to dye uptake throughout the lens and could then be compared between experimental conditions. This approach yielded a massive amount of data, best visualized using surface plots, but meaningful statistical analysis was beyond the scope of this study. The raw data were, therefore, not reported. However, this pilot study revealed topics for the focus of future research and eliminated certain ineffective routes of study.

In summary, we investigated the potential role of biomechanical stretching in driving lens transport. The findings generally indicated that lens transport was not significantly altered by stretching, at least on the timescales investigated in this study. It may be that any stretch-induced changes are very transient in nature, acting on the order of seconds rather than hours or days. Studying such brief events is infeasible with the current protocol owing to the lengthy processing of tissue to mount it on the stretcher; it seems likely that the biomechanical manipulations during the mounting process would overwhelm those of the stretching and would confound the effects. Still, it is certainly interesting that stretching may alter the pH of the lens’ anterior and that the distribution of MB within the lens is governed by some active process. Future work should focus on developing a more real-time monitoring of dye uptake, such as in calcium imaging.

## Data Availability

The original contributions presented in the study are included in the article/supplementary material. Further inquiries can be directed to the corresponding author.
